# An assessment of the food safety knowledge and attitudes of food handlers in hospitals

**DOI:** 10.1186/s12889-020-8430-5

**Published:** 2020-03-12

**Authors:** Lesiba A. Teffo, Frederick T. Tabit

**Affiliations:** Department of Life and Consumer Sciences, University of South Africa, Cnr Christiaan de Wet Road and Pioneer Avenue, Florida, Roodepoort, 1710 South Africa

**Keywords:** Food safety knowledge, Attitude, Handling practices, Food service employees, Hospitals

## Abstract

**Background:**

The possession of inadequate food safety knowledge (FSK) by food handlers poses a serious threat to food safety in service establishments. The aim of this research was to investigate factors that influenced the FSK and food safety attitudes (FSA) of employees involved in the preparation and/or the serving of food from nine hospitals in the Capricorn District Municipality (CDM) in Limpopo Province, South Africa.

**Method:**

Up to 210 individuals (18–65 years) who were employed in these hospitals, and who were involved in the preparation and serving of food to patients were purposefully selected. Data collection was by means of an interview using a questionnaire design for this study. The FSK and FSA scores of hospital food handlers were obtained by adding the correct response to FSK or FSA questions.

**Results:**

Only 29% of the hospital food handlers have attended a food safety-training course. Many food handlers were not knowledgeable on the correct temperature for handling foods, and on the correct minimum internal cooking temperature for poultry, seafood and egg. Only the minority of food handlers knew that *Salmonella* is the main foodborne bacteria pathogen mostly associated with poultry products (47.1%) and that food borne bacteria will grow quickly in food at a temperature of 37 °C (38.1%). Hospital food handlers with higher academic qualifications do not possess more FSK than those with lower academic qualifications. 51% of the hospital food handlers possessed a Satisfactory FSK while 10% possessed a Good FSK and 39% possessed an Inadequate FSK.

**Conclusion:**

More than 60% of the hospital food handlers possesses either Good FSK or Satisfactory FSK. Higher levels of education, experience in food handling and job position did not lead to better FSK outcome. All the hospital food handlers possess at least a Satisfactory FSA. There was a weak positive but significant correlation between the FSK and FSA of hospital food handlers. It is recommended all employees involved in food handlers be subjected to food safety training programmes on a regular basis irrespective of their academic, employment and training details.

## Background

The cooking and storage of food at incorrect temperatures and the cross-contamination of food due to unhygienic handling practices are regarded as the main causes of many foodborne disease outbreaks in food preparation and service facilities [1]. Factors such as poor personal hygiene and the procurement of food from unreliable sources have been found to contribute to foodborne disease outbreaks in food preparation and service facilities [2]. The possession of inadequate FSK by food handlers poses a serious threat to food safety in food preparation and service establishments such as hospitals [3]. The FSK, FSA and food handling practices of food handlers have always been a cause for concern over the years due to high incidences of foodborne disease outbreaks [4]. The possession of inadequate FSK by food handlers can translate in low level of food safety consciousness during food handling [5]. Therefore, all food handlers are required to possess adequate FSK and food handling skills to handle food hygienically during preparation and serving of to ensure that food is safe by the time it reaches the consumer [3]. Food handlers are required to avoid the contamination of food by microbes by maintaining high standards of food hygiene and sanitation at all times [6].

Many hospitals in South Africa contain food service units that are responsible for preparing and serving meals to patients in hospital wards. These food service units are expected to adhere to the ‘*Regulations governing general hygiene requirements for food premises, the transport of food and related matters ((R638 of 2018)*’ of the republic of South Africa. This regulation lays the framework for the implementation of food safety and makes provision for health inspectors to ascertain that food services premises comply with the law by having the necessary resources, conditions and infrastructure to ensure the safe handling of food and are handling food safely [7]. Some food service facilities implement the HACCP system as a mean to ensure the production of safe and quality foods [8]. Often, food handlers may lack the relevant food safety knowledge required for adequate implementation of proper hygiene processes during the preparation and serving of food in hospitals [9]. Furthermore, the lack of adequate infrastructure and proper sanitation facilities in some hospital may hinder the proper implementation of food safety measures in a hospital environment [10].

Foodborne disease outbreaks can cause morbidity and mortality of patients and workers in hospital and in the public leading to increased hospitalization cost for the public health department [11]. When there is a foodborne disease outbreak, the government incurs costs by funding health institutions to deal with the problem [12, 13]. An outbreak of foodborne disease outbreaks in hospitals can lead to service disruption, life threatening diseases and even death for anyone who is infected, especially the already vulnerable patients [14]. Inadequate food handling practice among food handlers have been found to be associated with low levels of food safety knowledge [15]. The food service facilities in hospitals in the Capricorn District Municipality procure raw food materials and prepare meals for hospitalised people. However, there is very little information is available on FSK, FSA and food handling practices of food service employees in hospitals in the Capricorn District, hence the purpose of this study, therefore, is to investigate factors that influence the FSK, FSA and food handling practices of food service employees in hospitals in the Capricorn District Municipality in Polokwane, Limpopo Province, South Africa.

## Methods

### The study area

This research project was conducted in the Capricorn District Municipality (CDM), which is located in the center of the Limpopo Province in South Africa. Limpopo Province is one of the nine provinces in South Africa. The Capricorn District Municipality has five local municipalities, which are Blouberg, Molemole, Lepelle-Nkumbi, Aganang and Polokwane, which is the capital city of Limpopo Province.

### Research design and sampling

A cross-sectional survey was conducted in which questionnaires were utilised to obtain data from hospital food handlers from nine government hospitals with food service units in the Capricorn District Municipality. Up to 210 individuals (18–65 years) who were employed in these hospitals, and who were involved in the preparation and serving of patients were purposefully selected based on their availability at their dedicated workstations in the hospitals.

### Research instrument

The data collection instrument was a questionnaire, which comprised of two sections: Socio-biographic section and FSK and FSA section. The reliability and validity of the different sections of the research instrument were determined and Cronbach’s α for the different constructs ranged from 0.689 to 0.821.

### Data collection

Prior to data collection, the permission to conduct the study was obtained from the Limpopo Provincial Department of Health and the University of South Africa provided the ethics clearance. Data collection was done by means of an interview with hospital food handlers after appointments to conduct an interview had been made with the hospital management. The interviews were conducted on a one-on-one basis and the questionnaire was filled in either by the hospital food handlers themselves or with the assistance of the principal researcher depending on the respondent’s level of literacy. Hospital food handlers were asked to sign a consent form to confirm their voluntary participation as well as their right to withdraw from the study if they so desired. The questionnaire of each respondent was coded to ensure anonymity and each interview session lasted about 20 min.

### Statistical analysis

The data collected were statistically analysed using SPSS software version 23. Descriptive statistics were used to summarise the variables of interest. Analysis of variance (ANOVA) was used to determine how hospital food handlers within socio-demographic groups differ in the response to questions while Partial Cross Tabulation (PCT) was used to see the proportion of responses.

The FSK and FSA scores of hospital food handlers were obtained by adding the correct response to FSK and FSA questions. The assessment of FSK scores out of 13 was conducted as follows: Scores of 1–6 = Inadequate FSK, Scores 7–9 = Satisfactory FSK and 10–13 = Good FSK. Similarly, the assessment of FSA scores out of 6 was conducted as follows: Scores of 1–2 = Inadequate FSA, Scores 3–4 = Satisfactory FSA and 5–6 = Good FSA. Statistical significance was identified at a 95% confidence level (*P* ≤ 0.05).

## Results

### Socio-demographic characteristics of hospital food handlers

Of the 210 hospital food handlers who participated in the study, 79%, the majority, were females while 20.5% were males. Regarding racial distribution, the vast majority of the hospital food handlers were Africans (99.5%), and the rest were whites (0.5%). No Coloured, no Indian, or Asian / other race groups participated in the study. The majority of hospital food handlers were between 18 and 35 years (68.8%) and single (64.8%), and only 31% were married. The rest were divorced, widowed or separated (4.2%). The majority of hospital food handlers (63.3%) had obtained qualifications higher than the high school Matric certificate, out of which, 33.3% had obtained a college certificate/diploma, 5.7% a higher certificate/diploma and 24.3% a bachelor’s degree (Table 1).

**Table 1:** Biographic information of hospital food handlers (*N* = 210)

Variables	Frequency (%)
Gender	
Female	167(79.5)
Male	43(20.5)
Race	
African	209(99.5)
White	1(0.5)
Indian	0
Coloured	0
Asian/others	0
Age	
18–25 years	73(34.8)
26–35 years	44(21)
36–45 years	50(23.8)
46–55 years	24(11.4)
56–65	19(9)
Marital status	
Single	136(64.8)
Married	65(31)
Divorced	3(1.4)
Widowed	4(1.9)
Separated	2(1)
Level of education	
Below matric certificate	24(11.4)
Matric certificate	53(25.2)
Certificate/Diploma	70(33.3)
Higher certificate/Higher diploma	12(5.7)
Bachelor’s degree/Postgraduate certificate	51(24.3)

### Employment and training details of hospital food handlers

The majority of the hospital food handlers were full-time employees (71%), while the others were either part-time (1.9%) or temporary employees (27.1%). Regarding their current employment position, the majority of the hospital food handlers were health care staff (70.5%), followed by chefs (16.2%), food service supervisors (5.7%), food service managers (4.8%) and support staff (2.9%). Most of the hospital food handlers (55.5%) had more than 4 years of work experience as a food handler, out of which 20.5% of them had between 5 and 7 years, while 10% had between 8 and 10 years and 24.8% had above 10 years. A huge majority of hospital food handlers (70%) earned R10000 (656.62$) or lower, out of which 28.1% earned below R5000 (328.31$) and 41.9% between R5001-R10000. Only a minority of hospital food handlers (27.6%) had attended a food safety-training course (Table 2).

**Table 2:** Employment and training details of hospital food handlers (*N* = 210)

Variables	Frequency (%)
Type of employment	
Full time	149(71)
Part time	4(1.9)
Temporal	57(27.1)
Current employment position	
Food service managers	10(4.8)
Food service supervisors	12(5.7)
Chef	34(16.2)
Nurses	148(70.5)
Support staff	6(2.9)
Experience in food handling practices	
Under 2 years	38(18)
2–4 years	56(26.7)
5–7 years	43(20.5)
8–10 years	21(10)
Above 10 years	52(24.8)
Income per month	
Below R5000	59(28.1)
R5001-R10000	88(41.9)
R10001-R15000	32(15.2)
R15001-R20000	17(8.1)
Above R20000	14(6.7)
Food Safety Training course	
Yes	58(27.6)
No	152(72.4)

### Knowledge on storage temperatures

The majority of hospital food handlers (59) correctly indicated 5 °C or lower as the correct temperature for receiving temperature control for safety (TCS) food. Similarly, only a few hospital food handlers (8.1%) correctly indicated 7 days as the correct maximum duration for which prepared ready-to-eat TCS food prepared in-house be stored at 5 °C. Only 31.9% of hospital food handlers correctly indicated ‘Thawing in the refrigerator’ as the best way to safely thaw frozen meat (Table 3). Hospital food handlers within the subgroups under the level of education and experience in food handling significantly (*p* ≤ 0.05) differ in the manner they responded to the question on the correct temperature for receiving TCS food. Similarly, Hospital food handlers within the subgroups under the level of education and food safety training attendance significantly (*p* ≤ 0.05) differ in the manner they responded to the question on the correct temperature for receiving TCS food. Hospital food handlers with higher levels of education and experience in food handling were not necessarily more knowledgeable in providing the correct responses to knowledge questions on the correct temperature for receiving TCS food (PCT1 and PCT2). Similarly, those with higher level of education and those with higher experience in food handling were not necessarily more knowledgeable in providing the correct responses to knowledge questions on the best way to safely thaw frozen meat (PCT3, and PCT5). The Chefs were more knowledgeable in providing the correct responses to the questions on the best way to safely thaw frozen meat (PCT4), while the Nurses were the least knowledgeable (Table 4).

**Table 3:** Hospital food handlers’ response to knowledge questions based on temperature control (*N* = 210)

Knowledge questions on receiving and storage of TCS foods and answer options	Frequency (%)
Which of the following is the correct temperature for receiving TCS food?	
0 °C or lower	16(7.6)
**5 °C or lower**	**124(59)**
7 °C or lower	33(15.7)
10 °C or lower	36(17.1)
Which of the following is the maximum duration for which prepared ready-to-eat TCS food prepared in-house is stored at 5 °C?	
3 days	167(79.5)
5 days	26(12.4)
**7 days**	**17(8.1)**
9 days	0(0)
Which of the following is the best way to safely thaw frozen meat?	
Thawing at room temperature	71(33.8)
**Thawing in the refrigerator**	**67(31.9)**
Thawing under a bowl of cold water	31(14.8)
Thawing by heating in the microwave	41(19.5)

**NB**: Correct answer indicated in bold

**Table 4:** ANOVA of hospital food handlers’ response to knowledge questions based on temperature control

	ANOVA between groups (***p***-value)			
Knowledge questions	Level of Education	Job position/description	Experience in food handling practices	Food safety training course attendance
Which of the following is the correct temperature for receiving TCS food?	**0.039**^**¥PCT 1**^	0.057	**0.006**^**¥PCT 2**^	0.403
Which of the following is the maximum duration for which prepared ready-to-eat TCS food prepared in-house is stored at 5 °C?	0.395	0.275	0.347	0.186
Which of the following is the best way to safely thaw frozen meat?	**0.000**^¥**PCT 3**^	**0.001**^¥**PCT 4**^	**0.000**^¥**PCT 5**^	0.074

**PCT 1**: Below metric (CA = 83.3%, WA = 16.7%), Matric certificate (CA = 47.2%, WA = 52.8.6%), Certificate/Diploma (CA = 57.1%, WA = 42.9%), Higher Certificate/Diploma (CA = 50%, WA = 50%), Bachelor degree and above (CA = 64.7%; WA = 35.3%)

**PCT 2**: Under 2 years (CA = 52.6%, WA = 47.4%), 2–4 years (CA = 41.1%; WA = 58.9%), 5–7 years (CA = 65.1%, WA = 34.9%), 8–10 years (CA = 71.4%, WA = 28.6%), Above 10 years (CA = 73.1%; WA = 26.9%)

**PCT 3:** Below metric (CA = 83.3%, WA = 16.7%), Matric certificate (CA = 26.4%, WA = 73.6%), Certificate/Diploma (CA = 21.4%, WA = 78.6%), Higher Certificate/Diploma (CA = 8.3%, WA = 91.7%), Bachelor degree and above (CA = 33.3%; WA = 66.7%)

**PCT 4**: Food service managers (CA = 50%, WA = 50%), Food service supervisors (CA = 41.7%, WA = 58.3%), Chef (CA = 58.8%, WA = 41.2%), Support staff (CA = 50%, WA = 50%), Nurses (CA = 23%; WA = 77%)

**PCT 5:** Under 2 years (CA = 39.5%, WA = 60.5%), 2–4 years (CA = 17.9%; WA = 82.1%), 5–7 years (CA = 20.9%, WA = 79.1%), 8–10 years (CA = 23.8%, WA = 76.2%), Above 10 years (CA = 53.8%; WA = 46.2%)

¥: Significance at *p* ≤ 0.05, *PCT* Partial Cross Tabulation, *CA* Correct Answer, *WA* Wrong Answer

### Knowledge on internal cooking temperature

Most of the hospital food handlers did not know the minimum internal cooking temperature for meat, poultry, seafood and eggs. Only 9.05% of hospital food handlers correctly indicated 74 °C for 15 s as the correct minimum internal cooking temperature for meat, poultry, and seafood. Similarly, only 17.6% of hospital food handlers correctly indicated 68 °C for 15 s as the correct minimum internal cooking temperature for eggs that will be hot-held for service. Furthermore, only 24.8% of hospital food handlers correctly indicated 68 °C for 15 s as the correct minimum internal cooking temperature requirement for ground beef. (Table 5). Hospital food handlers within the subgroups under the level of education, job position and years of experience as food handlers, significantly (*p* ≤ 0.05) differ in the manner they responded to the knowledge question on the internal cooking temperature requirement for eggs that will be hot-held for service. PCT1, PCT2 & PCT3 indicated that hospital food handlers with higher levels of education, years of experience as food handlers and within different job positions were not necessarily more knowledgeable in the provision of correct answers to knowledge questions regarding the correct minimum internal temperature for cooking eggs and the best way to safely thaw ground meat (Table 6).

**Table 5:** Hospital food handlers’ response to knowledge questions based on internal cooking temperature (*N* = 210)

Knowledge variables	Frequency (%)
Which of the following is the correct minimum internal cooking temperature requirement for meat, poultry and seafood?	
57 °C for 15 s	95(45.2)
63 °C for 15 s	76(36.2)
68 °C for 15 s	20(9.5)
**74 °C** for 15 s	**19(9.05)**
Which of the following is the correct minimum internal cooking temperature requirement for eggs that will be hot-held for service?	
57 °C for 15 s	100(47.6)
63 °C for 15 s	50(23.8)
**68 °C for 15 s**	**37(17.6)**
74 °C for 15 s	23(11.0)
Which of the following is the minimum internal cooking temperature requirement for ground beef?	
57 °C for 15 s	21(10.0)
63 °C for 15 s	43(20.5)
**68 °C for 15 s**	**52(24.8)**
74 °C for 15 s	94(44.8)

**Table 6:** ANOVA of hospital food handlers’ responses to knowledge questions based on internal cooking temperature (*N* = 210)

	ANOVA between groups (***p***-value)			
Knowledge questions	Level of education	Job position/ description	Experience in food handling practices	Food safety training course attendance
***Temperatures control***				
Which of the following is the correct minimum internal cooking temperature requirement for meat, poultry, and seafood?	0.464	0.271	0.249	0.379
Which of the following is the correct minimum internal cooking temperature requirement for eggs that will be hot-held for service?	**0.000**^**¥ PCT 1**^	**0.000**^¥**PCT 2**^	**0.062**^¥**PCT 3**^	0.524
Which of the following is the minimum internal cooking temperature requirement for ground beef?	0.446	0.547	0.966	0.742

**PCT 1**: Below metric (CA = 66.7%, WA = 33.3%), Matric certificate (CA = 11.3%, WA = 88.7%), Certificate/Diploma (CA = 8.6%, WA = 91.4%), Higher Certificate/Diploma (CA = 16.7%, WA = 83.3%), Bachelor degree and above (CA = 13.7%; WA = 86.3%)

**PCT 2:** Food service managers (CA = 0%, WA = 100%), Food service supervisors (CA = 33.3%, WA = 66.7%), Chef (CA = 41.2%, WA = 58.8%), Support staff (CA = 16.7%, WA = 83.3%), Nurses (CA = 12.2%; WA = 87.8%)

**PCT 3:** Under 2 years (CA = 13.2%, WA = 86.8%), 2–4 years (CA = 16.1%; WA = 83.9%), 5–7 years (CA = 9.3%, WA = 90.7%), 8–10 years (CA = 14.3%, WA = 85.7%), Above 10 years (CA = 30.8%; WA = 69.2%)

**¥**: Significance at *p* ≤ 0.05, *PCT* Partial Cross Tabulation, *CA* Correct Answer, *WA* Wrong Answer

### Safe food handling attitudes

After analyzing the variables involved, the majority of hospital food handlers had the correct FSA. Regarding the receiving and storage of food, up to 70.5% agreed that food stored at an incorrect temperature should always be discarded. Up to 70% indicated that they checked the temperature of refrigerators at least once per day while 87.6% indicated that they always separate raw and cooked food during storage. Regarding the hospital food handlers’ FSA towards food handling and contamination risks, up to 82.4% of hospital food handlers indicated they would not go to work and partake in food preparation when they had diarrhoea. Similarly 89.5% of hospital food handlers indicated that they continued to wash their hands during food preparation, even if others did not wash theirs. Up to 77.6% believed that their individual food handling practices could impact the food safety standards in their food preparation facilities. The vast majority, namely 94.8%, agreed that it is important to improve food handling practices to reduce the risk of foodborne illnesses (Table 7).

**Table 7:** Safe food handling attitudes of hospital food handlers

Attitude questions on Safe food handling and answer options		Frequency (%)
**Receiving and Storage**		
Do you believe that food stored at an incorrect temperature must always be discarded?	**Yes**	**148(70.5)**
	No	43(20.5)
	No idea	19(9.0)
Do you always check the temperature of refrigerators at least once per day?	**Yes**	**147(70.0)**
	No	53(25.2)
	No idea	10(4.8)
Do you always separate raw and cooked food during storage?	**Yes**	**184(87.6)**
	No	20(9.5)
	No idea	6(2.9)
**Food handling and contamination risks**		
Do you always avoid partaking in food preparation when you have diarrhoea?	**Yes**	**173(82.4)**
	No	33(15.7)
	No idea	4(1.9)
Do you always wash your hands during food preparation, even if others do not wash theirs?	**Yes**	**188(89.5)**
	No	19(9.0)
	No idea	3(1.4)
Do you think it is important to improve hygiene practices to reduce the risk of foodborne illnesses?	**Yes**	**199(94.8)**
	No	5(2.4)
	No idea	6(2.9)

**NB**: Correct attitude indicated in bold

### Knowledge on foodborne bacteria and diseases

The minority of hospital food handlers gave correct answers to the knowledge questions concerning foodborne bacteria and diseases. 47.1% correctly indicated Salmonella sp. as the main foodborne bacterial pathogen mostly associated with poultry products while 38.1% correctly indicated that foodborne bacteria will grow quickly in food that reaches a temperature of 37 °C. The vast majority of hospital food handlers (91.9%) correctly indicated diarrhoea as the most common symptom for food poisoning. Similarly, the majority of hospital food handlers (66.7%) correctly indicated that preschool-age children are at a greater risk of contracting foodborne illnesses because they have not built up strong immune systems. The majority of hospital food handlers (71.4%) correctly indicated that children, older people and pregnant women are also more vulnerable to foodborne diseases (Table 8).

**Table 8:** Hospital food handlers’ response to knowledge questions on food-borne pathogens and diseases (*N* = 210)

**Knowledge questions on food-borne pathogens and answer options**		**Frequency (%)**
Which of the following is the main foodborne bacteria pathogens, mostly associated with poultry products?	**Salmonella**	**99(47.1)**
	Staphylococcus	39(18.6)
	*E. coli*	20(9.5)
	Botulinum	8(3.8)
	Do not know	44(21.0)
Which of the following best explains what will happen to food borne bacteria in food at a temperature of 37 °C?	Die	29(13.8)
	Do not grow	41(19.5)
	**Grow quickly**	**80(38.1)**
	Grow slowly	28(13.3)
	Do not know	32(15.2)
**Knowledge questions on food-borne diseases and answer options**		**Frequency (%)**
Which of the following is the most common symptom for food poisoning?	Headache	6(2.9)
	**Diarrhoea**	**193(91.9)**
	Rash	3(1.4)
	Constipation	4(1.9)
	Do not know	4(1.9)
2.3.4. Which of the following best explains why are preschool-age children at a higher risk for foodborne illnesses?	**They have not built up strong immune systems**	**140(66.7)**
	They are more likely to spend time in a hospital	8(3.8)
	They are more likely to suffer allergic reactions	32(15.2)
	Their appetites have increased since birth	4(1.9)
	All of the above	26(12.4)
2.3.5. Which of the following groups of people are more vulnerable to foodborne diseases?	Children	31(14.8)
	Older people	5(2.4)
	Pregnant women	16(7.6)
	**All of the above**	**150(71.4)**
	I do not know	8(3.8)

**NB**: Correct answer indicated in bold

Hospital food handlers within the subgroups under level of education and employment position, differed significantly (*p* ≤ 0.05) in their response to knowledge questions on identifying the correct pathogen associated with poultry products and indicating what will happen to food borne bacteria in food exposed at a temperature of 37 °C. PCT 1 and PCT3 indicated that hospital food handlers with higher levels of education did not differ in their response to these knowledge questions. PCT 2 and PCT 4 indicated that food service managers and chef were knowledgeable to these knowledge questions while the food service supervisor, support staff and health care workers were less knowledgeable. Hospital food handlers within the subgroups under level of education and Food safety training course attendance, differed significantly (*p* ≤ 0.05) in their response to knowledge questions on identifying the correct reason why preschool-age children at a higher risk for foodborne illnesses. PCT 5 indicated that hospital food handlers with higher levels of education did not differ in their response to these knowledge questions compared to those with lower levels of education. PCT 6 indicated that those who have attended food safety training were knowledgeable to the knowledge questions than those who have not attended a food safety-training course (Table 9).

**Table 9:** ANOVA of hospital food handlers answers to knowledge questions on food-borne pathogens and diseases (*N* = 210)

	ANOVA between groups (***p***-value)			
Knowledge questions	Level of Education	Employment position	Experience in food handling practices	Food safety training course attendance
***Food-borne pathogens***				
Which of the following is the main foodborne bacteria pathogens mostly associated with poultry products?	**0.000¥**^**PCT 1**^	**0.002**^**¥PCT 2**^	0.097	0.119
Which of the following best explains what will happen to food borne bacteria in food at a temperature of 37 °C?	**0.000**^**¥PCT 3**^	**0.010**^**¥PCT 4**^	0.257	0.330
***Food-borne diseases***				
Which of the following is the most common symptom for food poisoning?	0.077	0.127	0.160	0.073
Which of the following best explains why are preschool-age children at a higher risk for foodborne illnesses?	**0.030**^**¥PCT 5**^	0.317	0.220	**0.043**^**¥PCT 6**^
Which of the following groups of people are more vulnerable to foodborne diseases?	0.113	0.769	0.320	0.104

**PCT 1**: Below metric (CA = 75%, WA = 25%), Matric certificate (CA = 34%, WA = 66%), Certificate/Diploma (CA = 32.9%, WA = 67.1%), Higher Certificate/Diploma (CA = 58.3%, WA = 41.7%), Bachelor degree and above (CA = 64.7%; WA = 35.3%)

**PCT 2**: Food service managers (CA = 70%, WA = 30%), Food service supervisors (CA = 33.3%; WA = 66.7%), Chef (CA = 85.3%, WA = 14.7%), Support staff (CA = 33.3%, WA = 66.7%), Nurses (CA = 41.7%; WA = 58.3%)

**PCT 3**: Below metric (CA = 4.2%, WA = 95.8%), Matric certificate (CA = 32.1%, WA = 67.9%), Certificate/Diploma (CA = 40%, WA = 60%), Higher Certificate/Diploma (CA = 33.3%, WA = 66.7%), Bachelor degree and above (CA = 58.8%; WA = 41.2%)

**PCT 4**: Food service managers (CA = 90%, WA = 10%), Food service supervisors (CA = 50%, WA = 50%), Chef (CA = 67.6%, WA = 32.4%), Support staff (CA = 33.3%, WA = 66.7%), Nurses (CA = 39.9%; WA = 60.1%)

**PCT 5**: Below metric (CA = 91.7%, WA = 8.3%), Matric certificate (CA = 56.6%, WA = 43.4%), Certificate/Diploma (CA = 61.4%, WA = 38.6%), Higher Certificate/Diploma (CA = 75%, WA = 25%), Bachelor degree and above (CA = 70.6%; WA = 29.4%)

**PCT 6**: Yes: (CA = 60.3%, WA = 39.7%), No (CA = 55.3%; WA = 44.7%)

¥: Significance at *p* ≤ 0.05, *PCT* Partial Cross Tabulation, *CA* Correct Answer, *WA* Wrong Answer

### Assessment of food safety knowledge and attitude

**Fig. 1:**
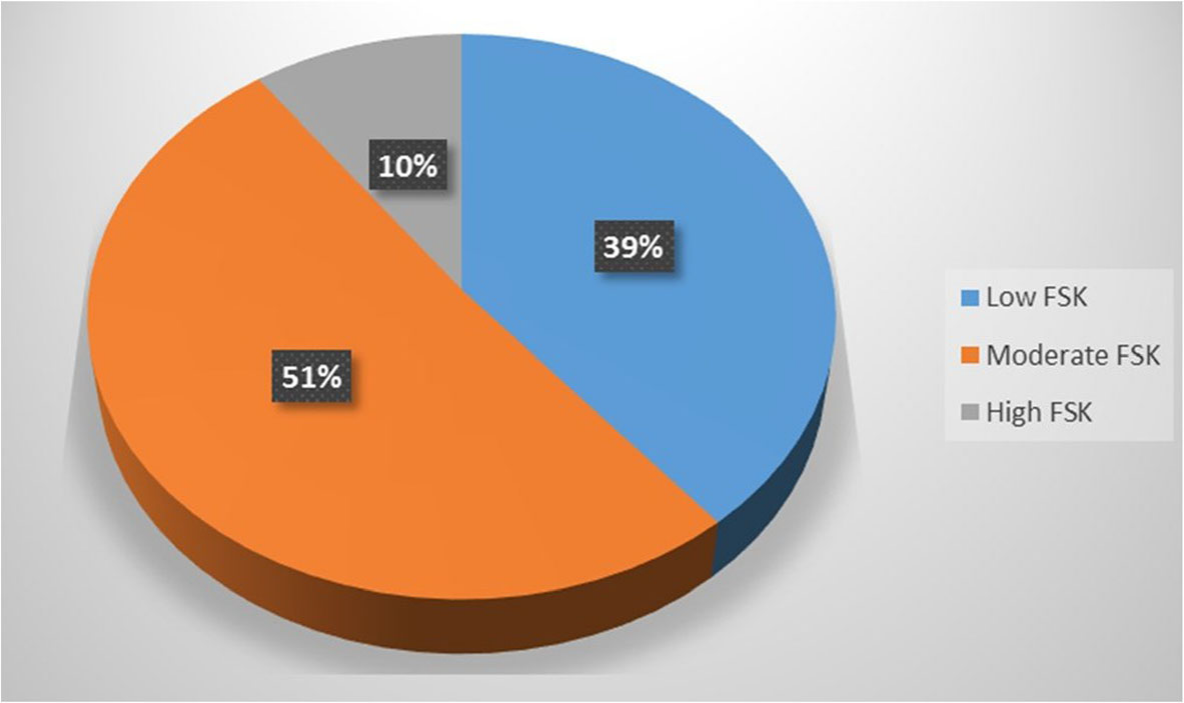
Food Safety Knowledge (FSK) assessment outcome of respondents (N = 210). IFSK = inadequate food safety knowledge (total Knowledge Score of 1–6). SFSK = Satisfactory food safety knowledge (total Knowledge Score of 7–9). GFSK = Good food safety knowledge (total Knowledge Score of 10–13)

**Fig. 2:**
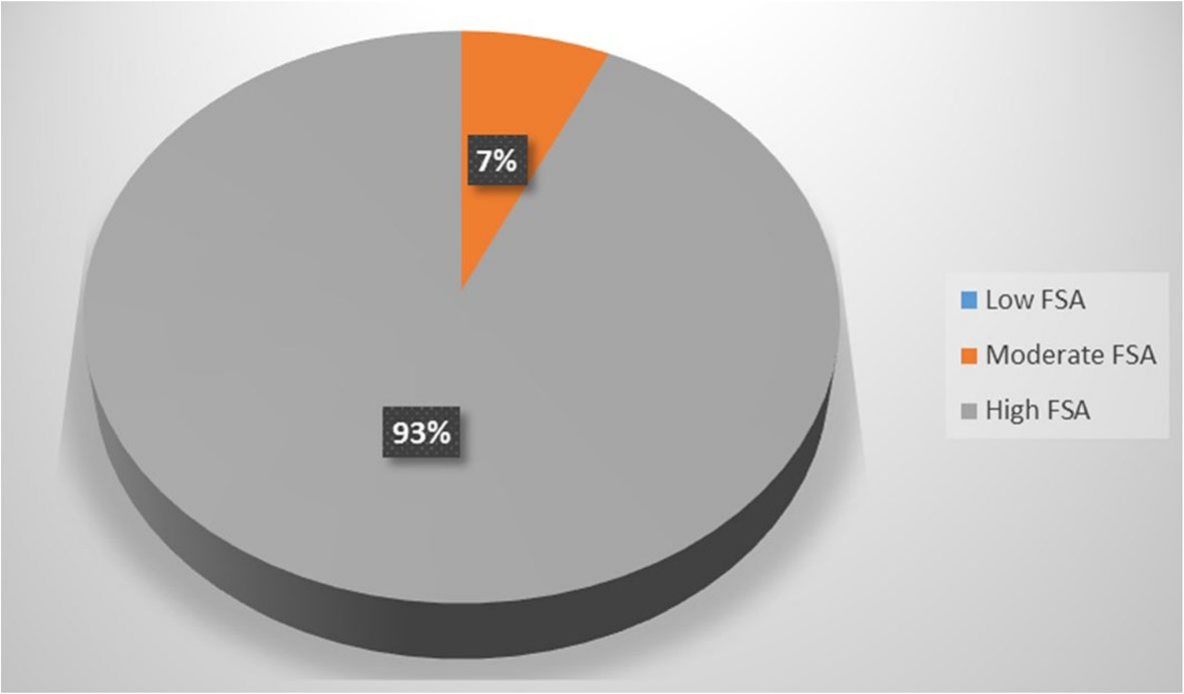
Food Safety Attitude (FSA) assessment outcome of respondents (N = 210). IFSA = inadequate food safety attitude (total attitude score of 1–2). SFSA = Satisfactory food safety attitude (total attitude score of 3–4). GFSK = Good food safety attitude (total attitude score of 5–6)

Overall, 51% of the hospital food handlers obtained a Satisfactory FSK outcome while 10% obtained a good FSK outcome and 39% obtained an Inadequate FSK outcome (Fig. 1). Hospital food handlers within the subgroups under level of education differed significantly (*p* ≤ 0.05) on their FSK assessment outcomes. However, food handlers with higher levels of education did not translate better FSK outcomes compared to those with lower levels of education. Similarly, hospital food handlers within the subgroups under experience in food handling practices differed significantly (*p* ≤ 0.05) on their FSK outcomes. However, food handlers with higher levels of experience in food handling practices did not translate to better FSK outcomes compared to those with lower levels of food handling experience. Hospital food handlers within the subgroups under job position/ description and food safety training course attendance did not differ significantly (*p* ≤ 0.05) on their safety knowledge assessment outcomes (Table 10). Up to 93% of the hospital food handlers obtained a Good FSA outcome while 7% obtained a Satisfactory FSA outcome and none obtained a Inadequate FSA outcome (Fig. 2). There was a weak positive (rho = 0.164) but significant (*p* ≤ 0.05) correlation between the FSK and FSA outcomes of hospital food handlers (Table 11).

**Table 10:** Difference in the food safety knowledge assessment outcomes of hospital food handlers within different socio-demographic groups (*N* = 210)

ANOVA between groups (***p***-value)			
Level of education	Job position/ description	Experience in food handling practices	Food safety training course attendance
**0.000**^**¥PCT 1**^	0.257	**0.003**^**¥PCT 2**^	0.838

¥: Significance at *p* ≤ 0.05, *PCT* Partial Cross Tabulation, Scores: 1–6 = Low FSK, 7–9 = Moderate KSK and 10–13 = High FSK

PCT1: Below Matric (Low FSK = 20.8%, Moderate FSK = 66.7%, High FSK = 12.5%) Matric Certificate (Low FSK = 50.6%, Moderate FSK = 39.6, High FSK = 3.8%), Certificate or Diploma (Low FSK = 48.6%, Moderate FSK = 44.3%, High FSK = 7.1%), Higher Certificate/Diploma (Low FSK = 8.3%, Moderate FSK =91.7%, High FSK = 0%), Bachelor’s Degree and above (Low FSK =23.5%, Moderate FSK = 54.9%, High FSK = 21.6%)

PCT2: Under 2 years (Low FSK = 55.3%, Moderate FSK = 36.8%, High FSK = 7.9%), 2–4 years (Low FSK = 37.5%, Moderate FSK = 53.6%, High FSK = 8.9%), 5–7 years (Low FSK = 41.9%, Moderate FSK = 53.5%, High FSK = 25.6%), 8–10 years (Low FSK = 42.9%, Moderate FSK =57.1%, High FSK = 0%), Above 10 years (Low FSK =25%, Moderate FSK = 53.9%, High FSK = 21.2%)

**Table 11:** Pearson correlation between the food safety knowledge and food safety attitude scores of respondents (*N* = 210)

Food safety knowledge score	0.164^a^
Food safety attitude score	

^a^ Correlation is significant at the 0.05 level (2-tailed)

## Discussion

### Demographics, employment and training details of hospital food handlers

The reason why the majority of hospital food handlers were females can be attributed to the fact that, In South Africa, women are more represented in all nursing and food service occupations compared to their male counterparts [16–18]. Furthermore, the Limpopo province is predominantly rural, hence very few people from other racial groups (Whites, Indians and Asians) in South Africa often prefer to live and work in rural areas [19]. The majority of hospital food handlers was found to be between 18 and 35 years old, single and were in their early years in their careers, getting involved with food handling in the hospitals [20–22].

The reason why the majority of hospital food handlers had obtained a qualification higher than a high school qualification (Matric) can be attributed to the fact that many young black South Africans, post 1994, have had increasingly more access to higher education than they did during the apartheid era in South Africa during which there was racial, political and economic discrimination against non-whites individuals from 1948 until the early 1990s [23]. The higher the level of education of food handlers, the easier it becomes for them to acquire FSK and skill through training [24]. The fact that the majority of the hospital food handlers were full-time permanent employees is beneficial for the hospitals considering that it ensures continuous improvement in FSK, FSA and the food handling skills of employees through continuous training and skills development without interruptions [25]. Regarding current employment positions, the majority of the hospital food handlers were health care staff and most probably nurses. Nurses determine the efficiency and effectiveness of hospital operations and constitute the majority of health care practitioners in most hospitals [26]. The reason why most of the hospital food handlers have been involved in food handling in their respective hospitals for four (4) years or more, is because most of the food handlers in hospitals are permanently employed on a full-time basis and permanent staff do not usually change jobs easily [27–29].

A huge majority of hospital food handlers earned below R10, 000 because many of the food handlers in hospitals are probably lower grade nurses and food service employees whose salaries often only increase over time through further training and the acquisition of professional experience [30]. The reason why only a minority of hospital food handlers have attended a food safety-training course maybe attributed be attributed to inadequate management support and commitment to the training of food handlers on food safety in hospitals [31, 32]. The lack of food hygiene training programmes could lead to inadequate FSK, which, in turn, could result in unsafe food handling practices [33–35].

### Knowledge on food handling temperatures

The majority of hospital food handlers correctly indicated 5 °C or lower as the right temperature for receiving TCS (temperature controlled for safety) food. This is extremely important, considering that temperature abuse can occur along the food chain if food handlers do not know the correct receiving temperatures of TCS foods [36]. Cooler temperatures can substantially reduce the rate at which food will deteriorate, because low temperatures slow down the growth of microorganisms in food thereby preventing food spoilage in hospitals. Contrarily, only a few hospital food handlers correctly indicated thawing in the refrigerator as the best way to safely thaw frozen meat as well as 7 days as the correct maximum duration for which prepared ready-to-eat TCS food prepared in-house could be stored at 5 °C. This can be attributed to the fact that most of the hospital food handlers have not received training on cold storage durations. Training programs geared toward food storage temperatures and duration can improve the food safety practice of food handlers. The ANOVA analysis showed that hospital food handlers with higher educational levels were not necessarily more knowledgeable in identifying the correct temperature for receiving TCS foods and correct FIFO procedures [37]. Hence, all food handlers in hospitals requires food safety training not only improve their FSK but also increase their self-efficacy in safe food handling practices and reduce their anxiety and stress levels [38] A study conducted in institutional catering facilities in Ghana also found low food safety knowledge of food handler on the storage of food in the danger zone and multiple freeze thaw cycles, and thawing of frozen food at room temperature [39].

### Knowledge on internal cooking temperatures

The vast majority of hospital food handlers did not know the minimum internal cooking temperature for meat, poultry, seafood and ground beef as well as correct minimum internal cooking temperature for eggs that will be hot held for service. This can be attributed to inadequate knowledge on internal cooking temperature of different food types [40, 41]. The misuse of time and temperature during the preparation and serving of food may lead to the contamination and proliferation of pathogens in food [42–44]. ANOVA indicated that even though hospital food handlers within the subgroups within level of education, job position and years of experience as food handlers significantly (*p* ≤ 0.05) differed significantly in their response to knowledge questions on the minimum internal cooking temperature for eggs that will be hot-held for service, food handlers with higher levels of education and experience as food handlers or a particular type of job position were not necessarily more knowledgeable on internal cooking temperature. This emphasise the fact that training on internal cooking temperature is essential [43]. The provision task specific lesson on internal cooking temperature can improve the food safety knowledge and improve food hygiene practices [45, 46].

### Safe food handling attitudes

Hospital food handlers possessed a positive FSA towards the discarding of food stored at incorrect temperatures and the checking of refrigerator temperatures at least once a day. These positive FSA ensure that foods that have been subjected to temperature abuse and which may contain high microbial loads are not processed for consumption in hospitals. It is important to check the temperature of refrigerators at least once a day considering that time-temperature abuses are the underlying cause of most foodborne disease outbreaks in food service establishments ([47]. Similarly, food handlers were found to possess the correct FSA to always separating raw and cooked foodstuffs during storage. This practice ensures the prevention of cross contamination between foods [48]. Most of the hospital food handlers also understood they should not go to work if suffering from diarrhoea and the importance of always washing their hands during food preparation. Their FSA towards seeking to improve on food handling practices was good. Good personal hygiene FSA contribute to the prevention of food borne pathogens being transmitted from the food handler to food [49]. Generally, the food safety knowledge level of food handlers has been found to have a positive effect on their food safety practices and attitudes [50]. However, the possession of positive FSA by food handlers has not always been found to translate into safe food handling practices [51].

### Knowledge on microbial hazards and foodborne diseases

The reason why only the minority of hospital food handlers (47,1%) correctly indicated *Salmonella* sp. as the main foodborne bacterial pathogen associated with poultry products may be attributed to the lack of microbial hazards knowledge by food handlers, which may be caused by lack of food safety education and training on microbial hazards in foods [52]. The fact that only a minority of hospital food handlers knew that pathogens in food will grow rapidly when food is subjected to temperatures of 37 °C is a concern regarding the correct handling of TCS food in hospitals and the prevention of microbial growth [51, 53]. The possession of inadequate knowledge of microbial hazards and critical temperature ranges by food handlers has been reported in many studies [54, 55]. This is further supported by the fact that food handlers within different subgroups under the level of education, job position and food safety training course attendance, significantly (*p* ≤ 0.05) differed on how they correctly indicated the main foodborne bacteria associated with poultry although they correctly stated that pathogens in food will multiply if the temperature of the food reaches 37 °C. However, higher levels of education, job position and food safety training courses did not enable the food handlers to answer these knowledge questions better than those who did not. The vast majority of hospital food handlers correctly indicated diarrhoea as the most common symptom for food poisoning. The vast majority of food handlers who participated in this study were nurses with more than 48 months of experience. This could be why the vast majority of food handlers in hospitals were knowledgeable on community health knowledge-based questions [54]. This can also explain why food handlers within the subgroups pertaining to levels of education, job position and food safety training course attendance, significantly (*p* ≤ 0.05) differed on how they correctly identified the group of people that are more vulnerable to food borne diseases. Higher levels of education, job position and their attendance at food safety training courses did not enable the food handlers to answer these questions more accurately.

### Food safety knowledge assessment

The majority of food handlers possessed a Satisfactory FSK and the fact that up 39% of hospital food handlers obtained an Inadequate FSK outcome implies hospital food handlers in these hospitals need continuous, and effective training on food safety measures [51, 54]. The possession of higher-level qualification and experience in food handling as well type of job description did not improve the overall FSK assessment outcomes of hospital food handlers hence justifying the notion of adequate FSK can mostly be attained through effective food safety training of food handlers [7]**.**

## Conclusion

The majority of respondents were knowledgeable on the symptoms of foodborne diseases as well as the vulnerable groups to foodborne diseases. The majority of respondents possessed a Satisfactory FSK outcome and good FSA outcome. Food handlers with higher levels of education, years of experience and job position did not necessarily possess better FSK outcomes. There was a weak positive but significant correlation between FSK and FSA outcomes. It is recommended that the hospital management ensures that that all hospital food handlers, irrespective of their level of education, years of food handling experience or job description, be subjected to continuous food safety training especially on handing and minimum internal cooking temperatures of TCS foods.

## Data Availability

The data that support the findings of this research are available from the corresponding author FT. These data are not publicly available at the moment but will be deposited into the University of South Africa’s data sharing platform which currently being instituted. However, upon a reasonable request and with permission from the University of South Africa, data can be obtained from the corresponding author.

## Abbreviations

ANOVA: Analysis of Variance
CDM: Capricorn District Municipality
FSA: Food Safety Attitudes
FSK: Food Safety Knowledge
HACCP: Hazard Analysis Critical Control Point
PCT: Partial Cross Tabulation
TCS: Temperature Control for Safety
